# Acute Mesenteric Ischemia in a COVID-19 Patient: Delay in Referral and Recommendation for Surgery

**DOI:** 10.1155/2021/1999931

**Published:** 2021-11-12

**Authors:** Mohammad Hossein Bagheripour, Mohammad Ali Zakeri

**Affiliations:** ^1^Department of General Surgery, Faculty of Medicine, Rafsanjan University of Medical Sciences, Rafsanjan, Iran; ^2^Social Determinants of Health Research Centre, Rafsanjan University of Medical Sciences, Rafsanjan, Iran; ^3^Non-Communicable Diseases Research Center, Rafsanjan University of Medical Sciences, Rafsanjan, Iran

## Abstract

Coronavirus (COVID-19) is more common with symptoms such as fever, dry cough, and shortness of breath. However, it may be associated with COVID-19-induced gastrointestinal (GI) symptoms including acute mesenteric ischemia (AMI). These conditions make the diagnosis of AMI challenging. Timely referral with correct diagnosis and attention to the uncommon symptoms of COVID-19 can play an important role in the management and treatment of AMI in COVID-19 patients. We present a patient with AMI due to thrombotic complications of COVID-19, who referred to the hospital too late and ignored the recommendation for abdominal surgery.

## 1. Introduction

COVID-19 started rapidly in China and spread around the world quickly. It became a global pandemic, creating a wave of fear, anger, and anxiety among patients and members of the community [1, 2]. Fever, dry cough, and shortness of breath are the most common symptoms of COVID-19. However, studies have reported that the incidence of gastrointestinal (GI) symptoms caused by COVID-19 occurred in up to two-thirds of patients with COVID-19 [3]. Symptoms include anorexia, nausea, vomiting, diarrhea, abdominal pain, and GI bleeding that may occur 1 to 2 days before fever and shortness of breath [4]. Asymptomatic COVID-19 patients and carriers who have no or fewer symptoms during the incubation period make disease diagnosis difficult. There are also a number of GI disorders associated with fever that make it difficult to differentiate between symptoms associated with COVID-19 and other diseases [5].

Acute gastrointestinal problems include AMI, which is characterized by a group of diseases that interrupt blood flow to different parts of the small intestine. AMI leads to ischemia and secondary inflammatory changes and, if left untreated, leads to intestinal necrosis with approximately 50% mortality. Early diagnosis and surgical intervention are the basis of treatment to reduce AMI mortality [6]. This report describes a 78-year-old man with COVID-19, who delayed seeking and undergoing abdominal surgery. The insights gained from this study can help us pay more attention to the gastrointestinal symptoms of COVID-19 patients and correctly identify the clinical features.

## 2. Case Presentation

A 78-year-old male patient (height: 173 cm, weight: 74 kg, and BMI: 24.7) referred to a public hospital for acute abdominal pain from three days ago after seven days of dyspnea and coughing. He had a history of coronary heart disease and percutaneous coronary intervention 6 years ago. His drug history was metoprolol 25 mg tablets twice per day *(bid)*, Nitrocontin 6.4 mg tablets twice per day *(bid)*, and aspirin 80 mg tablets once daily. The patient reported no history of trauma to his abdomen and no history of substance or alcohol abuse. At presentation, the pulse rate was 105 beats per minute (BPM), the blood pressure (BP) was 80/60 mm Hg, the body temperature (BT) was 38.7°C, and the respiratory rate (RR) was 20 breaths/min. The baseline electrocardiography (ECG) showed tachycardia with sinus rhythm and without myocardial infarction (MI) or other arrhythmias. The main symptom was a sudden onset of generalized abdominal pain with periumbilical predominance with 10/10 intensity. He claimed obstipation along with nausea and vomiting in this period. He could not eat during these three days. He suffered from coughing, dyspnea, and tachypnea before onset of the abdominal pain.

He was visited two times in outpatient centers and prescribed with pantoprazole 40 mg once daily, hyoscine 10 mg q8h, and painkillers (Advil) before referring to our hospital. On the last hours of the third day, he was admitted to the COVID-19 emergency department. On the examination of the abdomen, the main findings were abdominal distention with no audible bowel sound and also generalized abdominal tenderness and rebound tenderness. He had an obvious abdominal guarding. Digital rectal examination showed an empty rectal ampulla with no sign of mucosal sloughing. Respiratory sounds were nearly normal.

Main laboratory data were positive specific COVID-19 polymerase chain reaction (PCR) (based on a nasopharyngeal swab). Leuckocytosis (WBC = 11.200) and prerenal azotemia (BUN = 120 mg/dl and CR = 2.5 mg/dl) were seen in the patient. The results of the laboratory are presented in Table 1.

**Figure 1:**
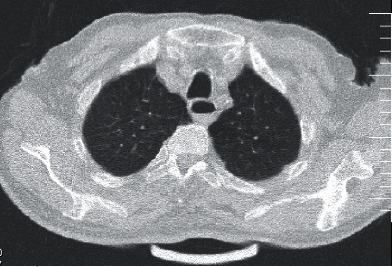
Male patient's chest CT.

**Figure 2:**
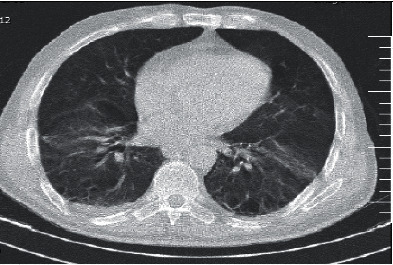
Male patient's chest CT.

**Figure 3:**
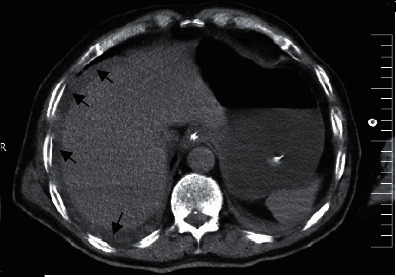
Male patient's abdominal CT (arrows: ascites).

**Figure 4:**
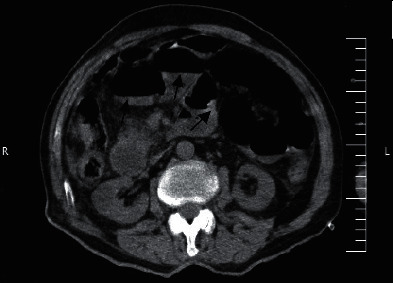
Male patient's abdominal CT (arrows: dilated small bowel loops).

Plane abdominal series radiographies and computed tomography (CT) of the chest were performed. A CT scan of the chest showed no definite findings of COVID-19 and cardiomegaly. There was no pleural effusion (Figures 1 and 2), but ascites and dilated small bowel loops could be seen in the few available axial cuts of the abdomen (Figures 3 and 4).

**Figure 5:**
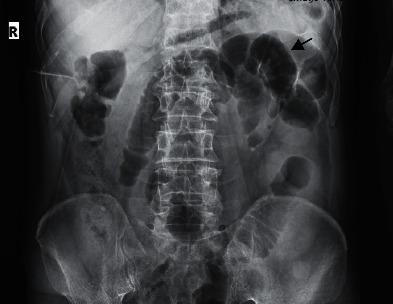
Male patient's supine abdominal X-ray.

**Figure 6:**
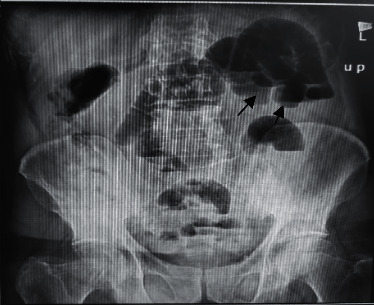
Male patient's upright abdominal X-ray.

Supine abdominal X-ray showed prominent dilated small bowel loops and the absence of a completely visible colon. Both findings were in favor of small bowel obstruction (Figure 5). Multiple air fluid levels were visible in the small bowel loops in upright abdominal X-ray, pointing at small bowel obstruction (Figure 6).

**Figure 7:**
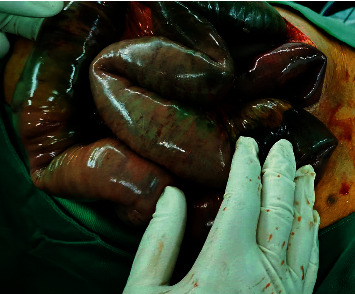
Photo of gangrenous small intestine of the patient.

After initial resuscitation with 2 liters of lactated ringer's solution and antibiotic therapy with empiric wide-spectrum prophylactic antibiotics (ceftriaxone 2 gr stat and metronidazole 500 mg stat), diagnostic laparotomy was performed. Massive brownish ascites and gangrenous small bowel and colon starting from 15 cm of ligamentum of Treitz to the middle part of the transverse colon were seen. Tissue decay has happened, and the intestine was about to perforate (Figure 7). These findings corresponded to acute mesenteric ischemia due to complete occlusion of the superior mesenteric artery for at least several hours. No therapeutic procedure could be performed, and the abdomen was closed. He died a couple of hours later in the intensive care unit (ICU).

## 3. Discussion

To the best of our knowledge, death of a patient with COVID-19 who had AMI and required emergency surgery was among the first cases of death due to delayed referral. Studies have shown that the mortality rate related to acute mesenteric arterial occlusion is very high. Necessary measures should be taken for the patient before the progression of ischemia to intestinal gangrene. Patient survival, on the other hand, depends on prompt diagnosis and revascularization [7].

Studies have shown that COVID-19 patients are more likely to develop AMI. Consistent with the present report, Chan et al. described a 73-year-old patient with ischemic colitis and showed that the patient died of cardiac arrest on day 5 of admission due to a hypercoagulable state. All inflammatory markers increased significantly in the patient [8]. Sehhat et al. described a 77-year-old patient and showed that impaired blood flow to the visceral-vascular system could cause life-threatening AMI [9]. Besides, Rashid et al. [10], Faridi et al. [11], Sevella et al. [12], and Shahid et al. [13] also confirmed these findings. Almeida et al. also described three patients with intestinal ischemia and pneumoperitoneum and showed that the pathogenicity of COVID-19 has been increasingly attributed to the systemic inflammatory response leading to intravascular disseminated coagulation [14].

Coagulopathy associated with COVID-19 has recently emerged as a major component of the disease and has led to high mortality [8]. Vartanoglu Aktokmakyan et al. showed the possibility of thrombotic events in patients with COVID-19. This observational study was conducted on 60 patients who underwent emergency surgery and showed that 5 out of 6 COVID-19 patients with acute abdomen (83.3%) underwent surgery due to AMI [15].

The pathogenesis of COVID-19 coagulation has not been fully understood yet. COVID-19 can cause a hypercoagulable state with acute inflammatory changes, which differs from disseminated intravascular coagulation (DIC) in that fibrinogen and D-dimer levels increase [16]. Tang found that disseminated intravascular coagulation and venous thromboembolism were the main causes of death in most COVID-19 patients [17]. However, it is not yet clear whether the coagulation disorders are a secondary response to the systemic inflammatory response or are developed directly by COVID-19. Studies on patients with acute COVID-19 have confirmed the presence of strokes and peripheral ischemia mediated by antiphospholipid antibodies [18].

However, several mechanisms or a combination of them may be responsible for AMI in COVID-19 patients. First, COVID-19 enters the cell through the receptor of the angiotensin-converting enzyme 2 (ACE2) in the alveoli and creates a severe form of infection with a systematic inflammatory response [19]. High levels of proinflammatory cytokines (interleukins 1 and 6 and interferon-g), known as the “cytokine storm,” have been reported in these patients [20]. Evidence has shown that inflammation activates coagulation, which in turn increases inflammatory activity, and there is a mutual relationship between inflammation and coagulation [21].

Saving of time is one of the most important challenges in the recovery of AMI patients that should be considered. A retrospective cohort study on 72 patients with AMI showed that a delay in operation (>6 hours after surgical consultation) and delay in surgical consultation (>24 hours after disease onset) were associated with increased mortality [22].

The patient presented in this report experienced acute gastrointestinal symptoms during 3 days, which caused his disease to become acute due to fear of COVID-19 and delayed referral. Fear and anxiety related to referral have been reported in different patients during the prevalence of COVID-19. This has caused acute problems and even death of some patients [23]. Studies have reported a high prevalence of fear and anxiety caused by COVID-19 [24, 25]. Physicians' attention to the mental and psychological condition of patients and involvement of the patients in the invasive procedures during the outbreak of COVID-19 are among the most important challenges facing the medical system that can have a direct impact on patient survival. In addition, paying attention to the warning signs and correct diagnostic methods are other issues that should be considered by physicians.

In addition to pulmonary symptoms, gastrointestinal symptoms are increasingly seen in COVID-19. Nausea/vomiting, diarrhea, abdominal pain, abdominal distension, or worsening of systemic conditions (such as sepsis) may be symptoms of patients with severe COVID-19 and AMI [26]. CT can help diagnose AMI. Dilated (>3 cm), thick-walled, and edematous bowel should be considered as signs of AMI. In advanced cases, pneumatosis intestinalis or portal venous gas suggests bowel ischemia. The presence of gas in the intestinal wall should be interpreted with caution because the presence of pneumatosis may be due to mechanical ventilation of the patients with severe COVID-19 [27]. Pirola et al. have described radiologic small bowel alterations including mesenteric ischemia in 50%, small bowel wall thickening in 16%, pneumatosis in 15%, intussusception in 13%, pneumoperitoneum in 3%, and paralytic ileus in 3% in 62 patients with COVID-19 [3].

There are challenges in the management of thromboembolic complications of COVID-19 due to the lack of high quality evidence of the efficacy and safety of various methods of prevention or treatment [28]. One of the suggested methods is thromboprophylaxis with low-molecular-weight (LMW) heparin [10]. Unlike chronic mesenteric ischemia, the treatment of acute arterial mesenteric ischemia requires a thorough bowel evaluation and revascularization by surgical means [7]. AMI can lead to ischemia and secondary inflammatory changes and, if left untreated, intestinal necrosis. Early diagnosis and surgical intervention reduce AMI mortality [6].

For timely intervention, healthcare providers must consider high indicators for recognizing life-threatening complications of COVID-19, such as AMI. On the other hand, information about the warning signs of acute problems of COVID-19 should be provided at the community level to prevent delayed referral of the patients, who should implement the necessary recommendations.

## 4. Conclusions

According to our information, this is one of the first reports of delayed referral of the patients with COVID-19, which has caused acute and fatal mesenteric ischemia in the patient. AMI should be suspected in the COVID-19 patients with abdominal pain. Early referral and rapid diagnosis among patients with clinical features of COVID-19 as well as attention to the mental state of the patients to participate in treatment are important factors that lead to patient survival. However, further studies are needed to understand the relationship between COVID-19 and the occurrence of AMI and their effects on patients.

## Figures and Tables

**Table 1:** The laboratory findings of the patient.

Variable (hematology test)		Normal reference range
White blood cell count (WBC)	11.200	4000–10000 ∗ 1000/cmm
Red cell count (RBC)	5.20	3.9–5.6 mil/cmm
Hemoglobin (Hb)	16.5	12–16 mmol/L
Hematocrit (HCT)	47.8	36–46%
Platelet count (PLAT)	146	150–450 ∗ 1000/cmm
Sodium (NA)	139	135–145 mEq/l
Potassium (K)	4.9	3.8–5 mEq/l
Blood urea nitrogen (BUN)	120	17–43 mg/dl
Creatinine (CR)	2.5	0.7–1.4 mg/dl
Total bilirubin	1.2	0.1–1.2 mg/dl
Direct bilirubin	0.6	0.1–0.3 mg/dl
C-reactive protein	Positive	Negative/positive
Amylase	593	Up to 110 U/L
Aspartate aminotransferase (AST or SGOT)	106	Up to 31 U/L
Alanine aminotransferase (ALT or SGPT)	76	Up to 31 U/L
Alkaline phosphatase (ALP)	206	64–306 U/L
D-dimer	301	Up to 0.5 mg/L

cmm: cells per cubic millimeter; mil/cmm: million cells/cells per cubic millimeter; mmol/L: millimoles per litre; mEq/L: milliequivalents per litre; mg/dL: milligrams per decilitre; U/L: units per litre; and mg/L: milligrams per litre.

## Data Availability

No data were used to support this study.
